# The Need for Psychological Support of Health Workers during the COVID-19 Pandemic and the Influence on Their Work

**DOI:** 10.3390/ijerph19158970

**Published:** 2022-07-23

**Authors:** Borja Nicolás Santana-López, Yeray Gabriel Santana-Padilla, María Desamparados Bernat-Adell, Jesús María González-Martín, Luciano Santana-Cabrera

**Affiliations:** 1 Intensive Care Unit, Hospital Universitario de Gran Canaria Doctor Negrín, 35010 Las Palmas de Gran Canaria, Spain; 2 Nursing Department, Universitat Jaume I, 12006 Castellón de la Plana, Spain; bernatm@enf.uji.es; 3 Emergency Surgery Unit, Complejo Hospitalario Universitario Insular Materno-Infantil de Gran Canaria, 35016 Las Palmas de Gran Canaria, Spain; yeraysantana@celp.es; 4 Research Support Unit, Complejo Hospitalario Universitario Insular Materno-Infantil de Gran Canaria, 35016 Las Palmas de Gran Canaria, Spain; josu.glez32@gmail.com; 5 Intensive Care Unit, Complejo Hospitalario Universitario Insular Materno-Infantil de Gran Canaria, 35016 Las Palmas de Gran Canaria, Spain; lsancabx@gmail.com

**Keywords:** mental health, health personnel, attitudes of health personnel, pandemics, COVID-19, emotions, work engagement

## Abstract

The aim of this research was to analyze how the need for psychological support of health workers (HCWs) influenced the beliefs, perceptions and attitudes towards their work during the COVID-19 pandemic and to predict the need of psychological assistance. A descriptive transversal study was conducted based on a self-administered questionnaire distributed to health professionals working in the Canary Islands, Spain. The data were analyzed using Pearson’s chi-squared test and the linear trend test. The correlation test between ordinal and frequency variables was applied using Kendall’s Tau B. Multiple logistic regression was used to predict dichotomous variables. The sample included 783 health professionals: 17.8% (*n* = 139) of them needed psychological or psychiatric support. Being redeployed to other services influenced the predisposition to request psychological help, and HCWs who required psychological support had more negative attitudes and perceptions towards their work. After five waves of COVID-19, these HCWs reported to be physically, psychologically and emotionally exhausted or even “burned out”; they did not feel supported by their institutions. The commitment of health personnel to fight against the COVID-19 pandemic decreased after the five waves, especially among professionals who required psychological support.

## 1. Introduction

Health professionals are considered highly vulnerable to mental health issues derived from the effects of the COVID-19 pandemic, including stress, anxiety, depression, emotional exhaustion, substance abuse and even post-traumatic stress disorder (PTSD) from the events experienced during the health crisis [1,2]. Systematic review studies conducted in the early stage of the pandemic support this hypothesis. Pappa et al. reported that almost two out of five healthcare workers (HCWs) suffered insomnia, and Vindegaard et al. stated that these workers had higher levels of anxiety, depression and sleep issues in comparison with the general population [3,4].

On the other hand, the working environment, task organization and working conditions are widely known factors that influence (negatively or positively) workers’ mental health and psychological well-being [5]. Zhou et al. highlighted the importance of the protective effects of organizational support to buffer the negative effects of work-related stressors [6]. These authors indicated that social and organizational support had independent predictive effects on the well-being of HCWs, with social support seeming more closely related to depression and anxiety and organizational support being more closely related to burnout [6].

According to a pre-pandemic study carried out in our country with 7539 nurses from 59 public hospitals of the Spanish national health system, half of them rated their work environment as unfavorable, one in five indicated that they would like to leave the hospital and more than the 20% of them showed high levels of burnout [7]. In line with an inform of 2019 from the Ministry of Health, Spain has one of the lowest nurse/patient ratios in the European Union (5.3 nurses/1000 people in Spain vs. 8.9/1000 in Europe) [8]. Therefore, it is highly plausible that these health personnel were already accustomed to high workloads and a lack of personnel, making the COVID-19 pandemic the perfect storm [8,9].

In addition to this, some authors hypothesized if the health personnel would have a duty to continue working during a catastrophic situation such as the current one, even when doing so implies an additional risk to themselves or their families [10,11]. Actually, it was expected that there would be a significant increase in the rates of absenteeism among them for this reason [12]. Nevertheless, during the first wave in March 2020, 70% of health professionals declared that they were willing to continue working, treating and caring for their patients despite the risk of infection due to the lack of personal protective equipment (PPE) [13]. Moreover, Tong et al. specified in their research that being single, having no children, possessing higher professional qualifications, working in a hospital and receiving employer-provided care training were predictive factors of nurses’ willingness to participate in the fight against COVID-19 [14].

This praiseworthy attitude of putting others before themselves in a time of uncertainty denotes the high commitment of these workers to their work, the health system and society [13,14,15]. Nonetheless, their willingness was not enough to guarantee patients proper quality of care. As stated in the research of Torrent-Ramos et al., almost half of the HCWs from a region of Spain perceived that the healthcare quality worsened at the beginning of the pandemic due to the work overload derived from the lack of personnel and materials [16].

After almost two and a half years since the first wave, some authors have begun to reassess the pandemic’s impact on the mental well-being of the same sample of HCWs at different chronological points, evidencing that their mental health worsened during the pandemic’s second year [17,18]. For example, during the third wave of COVID-19 in Canada, Crowe et al. described that the PTSD rates among critical care nurses (CNNs) had increased, even reaching levels up to 70%, when, before the health crisis, the maximum was around the 20% level [18]. In addition, although the intention of these nurses to leave the profession nearly tripled (from 8% to 22.4%), the authors objectified that the symptoms of depression, anxiety and stress remained similar compared to their previous study conducted in the early stage of the pandemic [18,19].

Based on the above, it could be postulated that an already precarious working condition, worsened by a prolonged health crisis, will have a negative impact on the physical, psychological and emotional wellbeing of HCWs and on their performance at their jobs [20,21]. This is likely to be the reason that many of these professionals have sought psychological therapy in order to process and overcome the emotions and the adverse circumstances experienced in their workplaces during this global health crisis. These elements could affect the engagement and job satisfaction of health professionals towards their institutions [22]. As other authors have noted, work engagement is influenced by many factors, including the feelings and attitudes towards that work [23].

The objective of this research is to analyze how the need for the psychological support of health workers influences the beliefs, perceptions and attitudes towards their work during the COVID-19 pandemic and to predict the need for psychological assistance.

## 2. Materials and Method

The present study was structured into two phases.

### 2.1. Phase One

First, a validated instrument used in a prior publication by this research group at the beginning of the pandemic in March 2020 was adapted to the epidemiological situation of the fifth wave to study the beliefs, attitudes and perceptions of the health personnel towards their work after applying the Delphi technique [13]. Furthermore, it should be noted that the original questionnaire on which this is based was developed and validated in 2009 by Damary et al., originally designed to study healthcare workers’ perceptions of their duty to work during an influenza pandemic [11].

During this process of adaptation, 14 health experts (from different professional categories and health services) agreed to collaborate with our group to review and evaluate the first updated version of the instrument generated by the researchers.

Thus, the initial dimensions of the original survey were kept; only some questions were reformulated, and new items from 3 validated scales were added and subsequently adapted (the Maslach Burnout Inventory, The COVID-19 Fear Scale and the Font-Roja job satisfaction scale) following the recommendations provided by the experts [24,25,26].

The first section was focused on the beliefs of healthcare personnel regarding the pandemic; the second was related to attitudes related to the work performed. The attitudes were divided into 2 groups of questions, Attitudes I and Attitudes II. The third one included questions of a personal nature, and the fourth included work-related questions regarding the interviewee, while the fifth section was related to their working perceptions.

Finally, the group of health experts agreed on the final version after evaluating its internal validity and controlling the coherence of the incorporated items. The reliability of the scale was assessed through the Cronbach’s alpha coefficient (α = 0.954) for the 78 included items. In addition to this, if we study the reliability of the dimensions independently, we obtained the following: beliefs (α = 0.619), attitudes (α = 0.940) and perceptions (α = 0.962).

### 2.2. Phase Two

A cross-sectional descriptive study was carried out through the questionnaire generated after the Delphi. This instrument was digitized into a “Google Forms” format and was latterly pretested by members of the research team to detect misunderstandings.

The electronic survey was distributed to the target population of the study: the health professionals who had worked during the COVID-19 pandemic at any public or private health institution in the Autonomous Community of the Canary Islands, Spain (doctors, medical interns, nurses, nursing interns, physiotherapists, auxiliary nursing care technicians, emergency medical technicians, radiology technicians and laboratory technicians). 

The questionnaire was sent by electronic means. In addition, in order to reach the target population, reference workers from hospitals, healthcare centers and socio-health centers were contacted, and these professionals managed the dissemination of the questionnaire. The study period was extended from 31 August to 23 September 2021, during the fifth wave of the COVID-19 pandemic in this region. 

As the instrument was self-administered, a brief explanation of the study was included in the first part, and express consent to participate in the research was requested. The participants were also informed that the questionnaire was anonymous, and the research team thanked them for their participation.

A total of 820 completed questionnaires were received, of which 37 were excluded for not giving their consent or responding inadequately to a control question (question designed to avoid automatic answers), leading to a final sample of 783 health professionals, which meant a sample error of 3.44% with a confidence level of 95%. The sample size was calculated considering the population of 22,882 health professionals currently working in the Autonomous Community (according to data from the regional government) [27]. 

In total, 17.8% (*n* = 139) of them reported that they required psychological or psychiatric support at some point during the pandemic by answering affirmatively to a dichotomous question included for this purpose.

### 2.3. Data Analyses

The selected subjects were split into two groups: those who reported needing psychological support during the pandemic (*n* = 139) and those who did not (*n* = 644). Differences between groups were analyzed for the five questions blocks.

A univariate analysis was performed by calculating the mean, standard deviation and median, and 25th and 75th percentiles were calculated for the quantitative variables. The Kolmogorov–Smirnov test was used to check the normality of the data. The frequencies and percentages were calculated for the qualitative variables.

A bivariate analysis was performed using Pearson’s chi-squared test to study the association between qualitative variables, and the linear trend test was applied to assess the relationship between ordinal and qualitative variables.

The correlation test between the ordinal and frequency variables was applied using Kendall’s Tau B, relating to having required psychological help or not, with the variables from the “perceptions” section, where the 4 possible answers were Infrequently, Monthly, Weekly and Daily.

To perform the multivariate binary regression analysis, two new variables were created with the values given to the Attitudes II and perceptions sections. In the section “Attitudes II”, the items that involved a positive attitude towards going to work were valued as one, and those that did not as zero. Regarding the “perceptions” section, the items were grouped into one variable, applying numerical values to the frequencies between 1 and 4, where higher values mean a positive feeling to continue working. Both variables allowed us to compare the groups.

Multiple logistic regression was used to predict the dichotomous variables. The area under the ROC curve was used to check the goodness of the model. The Stepwise: Forward–Backward method was used to obtain the optimal model to minimize the AIC (Akaike information criterion) error. A principal component analysis was carried out to check how the target variable was grouped according to the rest of the predictive variables. *p*-values lower than 0.05 were considered significant. The statistical analysis was performed with the collaboration of the Research Support Unit at the Maternal and Child Insular University Hospital Complex (CHUIMI) of Gran Canaria using the statistical program R Core Team 2020, version 3.6.3.

## 3. Results

### 3.1. The Sociodemographic and the Personal–Occupational Features of the Sample, According to Whether or Not They Required Psychological Help

It should be noted that most of the study subjects were women (*n* = 607; 77.5%) aged between 31 and 50 years old (*n* = 453; 57.9%). Nurses represented the greater number in terms of professional category (*n* = 403; 51.5%), and most of the subjects did not request psychological support (*n* = 644; 82.2%). No significant differences were found between these two groups. However, professionals under 30 years of age reported needing support in a significantly larger proportion than those over 50 (21.5% vs. 10.8%; *p* = 0.014). 

Furthermore, the proportion of professionals who needed psychological support was larger among those who had contracted COVID-19 (29.2% vs. 16.7%; *p* = 0.012), those who had to stay in home isolation (21.0% vs. 15.0%; *p* = 0.018), those who had leave or vacation periods cancelled (20.7% vs. 15.3%; *p* = 0.032) and those who had been transferred to other services (22.2% vs. 15.4%; *p* = 0.012). The results are included in Table A1.

### 3.2. Beliefs of the Health Professionals According to Whether or Not They Required Psychological Help and Their Attitudes towards Their Job (I and II) 

In general, no significant differences were found between these two groups. However, a larger proportion of the first group believed that a “second pandemic of post-traumatic stress” would affect health personnel due to the events experienced during the COVID-19 pandemic (85.6% vs. 72.2%; *p* = 0.002). A large proportion of professionals who needed psychological support claimed that they would not attend their job in case a family member died of COVID-19 (27.6% vs. 17.2%; *p* = 0.026).

Additionally, they disagreed with the following statements: “All health workers have the duty to work during the pandemic, even if there is a greater risk to their health” (56.8% vs. 46.3%; *p* = 0.015), “People who refused to work during this time of health crisis should be sanctioned in some way” (64.0% vs. 53.3%; *p* = 0.013) and “Responsibility at work is above family duties” (59.0% vs. 44.6%; *p* = 0.001). Moreover, they were less willing to renounce leave or vacation periods in case they were required to work (59.0% vs. 44.6%; *p* = 0.001). The results are included in Table A2. 

### 3.3. Working Perceptions of the Health Professionals According to Whether or Not They Required Psychological Help and Correlation for Ordinal and Frequency Variables Using Kendall’s Tau B

The health professionals who needed psychological support reported a higher proportion of emotional exhaustion (46.8% vs. 18.3%), physical and mental tiredness (45.3% vs. 22.2%) and a feeling of being “burned out” by their job (29.5% vs. 15.2%) (*p* < 0.001).

Moreover, they felt less satisfied with their job (19.4% vs. 36.0%), claimed to be at the limit of their endurance (18.0% vs. 6.4%), failed to stop thinking about work during their free time (13.7% vs. 22.8%) and had trouble sleeping since the beginning of the pandemic (21.6% vs. 8.1%) (*p* < 0.001).

It should be noted that, although they perceived their work as having a positive influence on other people’s lives (49.6% vs. 56.8%) (*p* < 0.001) and claimed that they would choose their profession again (52.5% vs. 67.4%) (*p* = 0.003), their proportion was lower than that of professionals who did not need psychological support. 

In addition, these HCWs reported that they felt infrequently supported by their superiors (35.3% vs. 23.9%; *p* = 0.015) and their institutions (56.1% vs. 42.9%; *p* = 0.009). 

Finally, we found a moderate correlation (0.40–0.59) in asking for psychological support between feeling emotionally exhausted due to work (Gamma = 0.523) and feeling physically and psychologically exhausted when finishing the working day (Gamma = 0.443). The results are shown in Table A3.

### 3.4. Logistic Regression to Predict The Target Variable (Needing Psychological Assistance)

A multiple logistic regression was performed to predict the target variable based on the beliefs, attitudes and perceptions and adjusting for age, gender and contracting COVID-19.

#### 3.4.1. Logistic Regression to Predict Psychological Support Based on Beliefs

Related to “beliefs”, the model explains that age is a protective factor for needing psychological or psychiatric support (OR = 0.97, (95% CI: 0.96–0.99); *p* = 0.001). Getting infected with COVID-19 is a risk factor compared to those who had not contracted the disease (OR = 2.06, (95% CI: 1.13–3.64); *p* = 0.015). Answering YES to “Have you lacked the appropriate PPE at any time during the pandemic?” is a risk factor with respect to those who answered NO (marginally significant result, OR = 1.43, (95% CI: 0.95–2.20); *p* = 0.086), and answering YES to “Do you think there will be a second “post-traumatic stress pandemic” among healthcare personnel due to the events experienced during the COVID-19 pandemic?” is a risk factor with respect to those who answered NO (OR = 3.81, (95% CI: 1.65–11.09); *p* = 0.005). Gender did not have a significant influence (Table 1).

#### 3.4.2. Logistic Regression to Predict Psychological Support Based on Attitudes

When exploring for the best model for “Attitudes I”, age was again a protective factor for needing psychological or psychiatric support (OR = 0.98, (95% CI: 0.96–1); *p* = 0.028), contracting COVID-19 was also a risk factor when compared to those who had not contracted the disease (OR = 1.99, (95% CI: 1.01–3.77); *p* = 0.039) and gender also showed no influence.

On the other hand, the items “If you were asked to take on additional tasks for which you had not been trained/educated” (OR = 0.46, (95% CI: 0.27–0.78); *p* = 0.004), “If you received some incentives from the institution” (OR = 2.16, (95% CI: 1.17–3.92); *p* = 0.012) and “If you were asked to work more hours” (OR = 1.94, (95% CI: 1.13–3.32); *p* = 0.015) were not significant factors for requesting psychological support (Table 2).

Regarding the block “Attitudes II”, the best regression model showed that age is a protective factor for needing psychological or psychiatric support (OR = 0.98, (95% CI: 0.97–1); *p* = 0.057)*,* contracting COVID-19 is a risk factor compared to those who had not contracted it (OR = 2.07, (95% CI: 1.14–3.66); *p* = 0.014) and gender did not have an influence. The model was repeated to analyze the variables of the block Attitudes II. 

This model was completed with the following variables: answering DISAGREE to “Those who refuse to work during this health crisis should be sanctioned in some way” is a risk factor with respect to those who answered AGREE (OR = 1.47, (95% CI: 1–2.18); *p* = 0.054), answering DISAGREE to “I would be willing to voluntarily give up my days off or vacation time to continue working if required by my unit” is a risk factor with respect to those who answered AGREE (OR = 1.66, (95% CI: 1.14–2.44); *p* = 0.009) and answering DISAGREE to “Working during the pandemic has been the most important challenge I have faced during my working life” is a protective factor with respect to those who answered AGREE (OR = 0.64, (95% CI: 0.36–1.08); *p* = 0.112). Although the result is not significant, this variable helps predict the target variable (Table 3).

#### 3.4.3. Logistic Regression to Predict Psychological Support Based on Perceptions

The results of exploring the best model for “perceptions” indicated again that age was a protective factor for needing psychological support (OR = 0.98, (95% CI: 0.96–1); *p* = 0.035), contracting COVID-19 was a risk factor (OR = 2.34, (95% CI: 1.22–4.35); *p* = 0.008) and gender did not show any influence.

In this case, the perceptions that helped to predict the target variable were as follows: “Because of my job I feel emotionally exhausted” (OR = 12.71, (95% CI: 4.76–40.93); *p* < 0.001), “Through my work I feel that I am positively influencing other people’s lives” (OR = 2.29, (95% CI: 1.16–4.45); *p* = 0.015), “At work I feel that I am at the limit of my possibilities” (OR = 0.49, (95% CI: 0.26–0.88); *p* = 0.0018) and “Despite everything, the pandemic has not ruined my vocation. If I could go back, I would choose my profession again” (OR = 2.03, (95% CI: 1.09–3.72); *p* = 0.023) (Table 4).

### 3.5. Multivariate Regression Analysis

When exploring the perceptions and attitudes towards work, the multivariate regression analysis showed that the attitudes and perception variables represented the mean values of 5.84 ± 0.061 and 47.09 ± 0.294, respectively. Then, the HCWs who did not require psychological support had more positive attitudes (5.93; *p* = 0.001) and positive perceptions (47.98; *p* < 0.001) towards their work (Table 5).

### 3.6. Binary Logistic Regression

Binary logistic regression indicates that the variable that most influences requesting psychological help is being redeployed to other units. Professionals who were relocated had a 1663 times higher risk of needing psychological help than those who were not (Table 6).

## 4. Discussion

The results of our research offer insight into the way the recent health crisis has influenced the mental health of healthcare professionals in the Canary Islands, where almost 20% of them required psychological or psychiatric support during the pandemic. Other authors in different continents reported similar results; for example, Kang et al. found that 17.5% of professionals received psychological therapy during the first two months of 2020 in Wuhan, China [28]. However, Alvarado et al. affirmed that only one-third of those who reported needing psychological help ended up receiving it [29].

This last finding is key to highlight the problem that we have identified here, because it is probably the case that many professionals may need this assistance but they do not request it. Stojanov et al. described that HCWs preferred to deal with these kinds of problems on their own rather than asking for psychological support, despite three out of five of them confessing that their mental state had worsened since the pandemic began [30]. Further studies are needed to help us determine other variables that may be part of this construct that we have not yet measured.

In our study, the larger proportion of professionals needing psychological support was found among younger professionals and those who had contracted COVID-19, those who had to be isolated and those who had leave or vacation periods cancelled. Such a profile agrees with the characteristics of professionals at a higher risk of psychological distress during the pandemic published by Kisley et al. in 2020 [31].

We verified that being redeployed to other services increased the request for psychological help. Tan et al. expressed, in their research that being redeployed was associated with higher scores in the Oldenburg Burnout Inventory [32].

According to our results, working with patients with COVID-19 is not always associated with the need for psychological support. We found similarities to the research of Cai et al., who compared the psychological impact of the pandemic on first- vs. second-line health personnel; despite the fact that professionals working with COVID-19 patients presented higher incidences of anxiety, depression and insomnia, no significant differences were found in suicidal ideation or active searching for psychiatric support [33].

In relation to their beliefs, there was a consensus among the participants in the affirmation that “There will be a “second pandemic” affecting the mental health of the healthcare professionals”. Studies conducted with health personnel working during the SARS outbreak in 2003 support this result, since more than 30% of health workers treating patients infected with SARS reported significantly higher levels of emotional exhaustion in comparison with health workers who did not treat these patients and non-sanitation workers up to two years after the outbreak [34,35,36].

It is important to emphasize that most professionals considered that the COVID-19 pandemic revealed the shortcomings of the national health system and that a reorganization of its structure after the health crisis is necessary. This situation has led authors such as García-Basteiro et al. to analyze how it was possible for a country such as Spain to suffer so greatly from the consequences of the COVID-19 pandemic when its health system was considered to be one of the best in the world [37]. They concluded that the Spanish National Health System was noticeably deteriorated by cuts in public health expenditure following the 2008 financial crisis, and since then, health professionals have been dealing with a high burden of care, resource and staff shortage and temporary employment conditions [9,37].

In connection with this, even though the Health Services of the Autonomous Community of the Canary Islands increased the number of healthcare professionals they hired during 2021 by 130% compared to 2018, almost the entire sample agreed with the affirmation that “Having more staff would have helped reduce the work overload during the pandemic” [27,38]. As mentioned before, Spain has one of the lowest nurse/patient ratios in Europe, and the Canary Islands is among the autonomous communities with the worst numbers (3.3 nurses for every 1000 patients) [39]. The WHO estimates that there will be a deficit of approximately 18 million health workers by 2030 and, to avoid this situation and guarantee quality health care to the population, urges countries to apply different policies such as, among many others, investing in the health workforce, promoting decent working conditions and enhancing the safety and protection of health personnel [40]. 

Regarding attitudes, up to one in five professionals who needed psychological support said that they would probably not go to work if a family member had died from COVID-19, and almost every one of them considered that their family duties were more important than their job responsibilities. Similar results were reported in previous studies, which supports the hypothesis that family is a determining factor in these workers’ decisions to continue with their job or not [11,12,13,41]. 

In the initial stages of the pandemic, according to our study in 2020, the vast majority of the health professionals were willing to work, despite the fact that doing so was a risk to their own health [13]. This commitment decreased after the five waves of COVID-19 among the HCWs in general but especially among those who required psychological help; almost 6 out of 10 of them disagreed with the following statement: “All health workers have the duty to work during the pandemic, even if there is a greater risk to their health”. Ke et al. described in their research that the Chinese nurses who were unwilling to work during the pandemic experienced more levels of burnout, anxiety, depression and fear in comparison with the nurses who were willing to work [42]. 

Related to perceptions, the health personnel who needed psychological support felt physically, psychologically and emotionally exhausted; at the limits of their endurance at work; less satisfied with their job and even “burned out”. They did not feel supported by their direct managers and felt undervalued by their institutions. In addition, they had had difficulty sleeping since the start of the health crisis and failed to stop thinking about work in their free time. Despite this, a high proportion would choose their profession again. These results agree with those of other authors; the pandemic has negatively influenced health professionals’ working conditions and, consequently, their emotional well-being [43,44,45,46,47]. 

To conclude, it is also worth noting that, during the lockdowns and the de-escalation processes of the first and second pandemic waves in our country (March–October 2020), sick leave due to mental issues increased by 30% [48]. In addition, according to the 2020 report of the Observatory of Occupational Diseases, workers from the health and social services areas requested the most amount of sick leave due to mental health issues, representing almost 30% of the total [49]. 

### Limitations

The main limitations of this study were the results of the type of sampling performed. First, there was the possibility that the HCWs who responded were the most motivated ones and those who had the worst experiences during the pandemic and were thus the most driven to express their feelings by responding to the questionnaire.

Second, we must be cautious when interpreting the findings regarding the relationship between mental health problems and certain work environments. This should be studied more deeply in the future. 

Third, prior to distributing the survey to the target population, we calculated that we needed a minimum sample size of 585 to extract reliable conclusions, with a sampling error of 4% and a confidence index of 95% (basing this calculation on the available data of the number of health workers currently working in this region). Unquestionably, when splitting the sample into both groups, we could still extract consistent conclusions using the group who did not need psychological support (585 < 644). On the other hand, currently, no official available data state the total number of health professionals who have received psychological assistance. For this reason, we cannot verify if we reached the minimum sample size to be able to treat them as a group. However, this fact is relevant, because our study highlights how many professionals have attended therapy during the COVID-19 pandemic. 

Given that this study was conducted in the outermost regions of the Canary Islands, it is difficult to extrapolate our conclusions to other regions, because this wave did not have the same impact. However, this study offers insight into the emotional states and perceptions of HCWs. 

## 5. Conclusions 

This crisis has influenced the mental health of healthcare professionals, where a significant percentage required psychological or psychiatric support during the pandemic, although it is probably the case that many professionals who needed it did not request it. The factor that influenced requesting psychological help to the greatest extent was being relocated to other services.

Gender is not a factor that determines the request for psychological support; nevertheless, the youngest HCWs were the ones who requested it the most, and those who had contracted COVID-19 were at risk of not asking for psychological support when exploring their attitudes, perceptions and beliefs.

It has been shown that commitment decreased after the five waves of the pandemic, especially in professionals who required psychological support. HCWs who required psychological support had more negative attitudes and negative perceptions towards their work, implying that the quality of treatment and care that patients could receive would be much lower, directly affecting their safety.

## 6. Proposals for the Future

This study was carried out after a year and a half of the pandemic (September 2021), a situation that seems far from improving. The rebound in new cases and virus strains could lead to new pandemic waves. Therefore, it is to be expected that, if the current working conditions continue, an increasing number of health professionals will interrupt their work to take care of their mental health.

Our results may be useful in managing human resources in health care and improving them, both in quantity and quality, in relation to the experiences and skills necessary to work in crisis situations.

It also opens the door for future studies to assess engagement in health care settings and improve access to request psychological support.

## Figures and Tables

**Table 1:** Logistic regression to predict psychological support based on beliefs.

Variables			Univariate Analysis						Multivariate Analysis (Optimal Model)				
			*n*	β	EE	OR	CI 95%	*p*	β	EE	OR	CI 95%	*p*
Demographics	Intercept								−2.09	0.6	0.124	0.03−0.38	<0.001
	Gender: Male		783	−0.12	0.23	0.89	0.56–1.38	0.615	-	-	-	-	-
	Age		783	−0.02	0.01	0.98	0.96–0.99	0.006	−0.02	0.01	0.977	0.96−0.99	0.011
	Contracting COVID-19		783	0.72	0.29	2.06	1.14–3.59	0.013	0.72	0.3	2.064	1.13−3.64	0.015
Beliefs	Have you lacked the appropriate PPE at any time during the pandemic?	YES	783	0.43	0.21	1.54	1.03–2.35	0.038	0.36	0.21	1.439	0.95−2.20	0.086
	Do you think there will be a second “post-traumatic stress pandemic” among healthcare personnel due to the events experienced during the COVID-19 pandemic?	NO				1 (ref)					1 (ref)		
		DON’T KNOW	783	0.84	0.54	2.317	0.85–7.37	0.118	0.85	0.54	2.333	0.85−7.46	0.118
		YES	783	1.38	0.47	3.992	1.74−11.55	0.003	1.34	0.48	3.815	1.65−11.09	0.005
AIC: 714.09. AUC ROC: 0.64													

Note. Logistic regression based on BELIEFS. *p*-value < 0.05.

**Table 2:** Logistic regression to predict psychological support based on Attitudes I.

Variables			Univariate Analysis						Multivariate Analysis (Optimal Model)				
			*n*	β	EE	OR	CI 95%	*p*	β	EE	OR	CI 95%	*p*
Demographics	Intercept								−0.57	0.49	0.57	0.22−1.47	0.242
	Gender: Male		609	−0.02	0.24	0.98	0.6–1.56	0.922	-	-	-	-	-
	Age		609	−0.02	0.01	0.98	0.96–1	0.067	−0.02	0.01	0.98	0.96−1	0.028
	Contracting COVID-19		609	0.67	0.32	1.96	1.03–3.59	0.034	0.69	0.33	1.99	1.01−3.77	0.039
Attitudes I	If you were asked to take on additional tasks for which you had not been trained/educated	PROBABLY	1 (ref)						1 (ref)				
		I DO NOT KNOW	609	−0.17	0.29	0.84	0.47–1.48	0.55	−0.3	0.31	0.74	0.40–1.34	0.324
		NOT PROBABLY	609	−0.39	0.24	0.679	0.42–1.09	0.111	−0.78	0.27	0.46	0.27–0.78	0.004
	If you received some incentives from the institution	PROBABLY	1 (ref)						1 (ref)				
		I DO NOT KNOW	609	0.19	0.29	1.207	0.66–2.10	0.52	0.24	0.31	1.27	0.67–2.31	0.444
		NOT PROBABLY	609	0.81	0.27	2.255	1.30–3.82	0.003	0.77	0.31	2.16	1.17–3.92	0.012
	If you were asked to work more hours	PROBABLY	1 (ref)						1 (ref)				
		I DO NOT KNOW	609	0.28	0.28	1.328	0.75–2.28	0.316	0.34	0.3	1.41	0.77–2.54	0.258
		NOT PROBABLY	609	0.62	0.24	1.861	1.16–2.96	0.009	0.66	0.27	1.94	1.13–3.32	0.015
	If a family member died from COVID-19	PROBABLY	1 (ref)						1 (ref)				
		I DO NOT KNOW	609	−0.17	0.27	0.846	0.48–1.42	0.537	−0.36	0.29	0.7	0.39–1.21	0.207
		NOT PROBABLY	609	0.61	0.25	1.845	1.11–3.02	0.016	0.3	0.28	1.35	0.77–2.33	0.288
AIC: 570.18. AUC ROC: 0.659													

Note. Logistic regression based on Attitudes I. *p*-value < 0.05. Records that have all the complete data were selected: 609.

**Table 3:** Logistic regression to predict psychological support based on Attitudes II.

Variables			Univariate Analysis						Multivariate Analysis (Optimal Model)				
			*n*	β	EE	OR	CI 95%	*p*	β	EE	OR	CI 95%	*p*
Demographics	Intercept								−1.31	0.43	0.27	0.11–0.62	0.002
	Gender: Male		783	−0.12	0.23	0.89	0.56–1.38	0.615	-	-	-	-	-
	Age		783	−0.02	0.01	0.98	0.96–0.99	0.006	−0.02	0.01	0.98	0.97–1	0.057
	Contracting COVID-19		783	0.72	0.29	2.06	1.14–3.59	0.013	0.73	0.3	2.07	1.14–3.66	0.014
Attitudes II	Those who refuse to work during this health crisis should be sanctioned in some way	DISAGREE	783	0.45	0.19	1.56	1.07–2.3	0.021	0.38	0.2	1.47	1–2.18	0.054
	I would be willing to voluntarily give up my days off or vacation time to continue working if required by my unit	DISAGREE	783	0.58	0.19	1.79	1.24–2.61	0.002	0.51	0.2	1.66	1.14–2.44	0.009
	Working during the pandemic has been the most important challenge I have faced during my working life	DISAGREE	783	−0.39	0.27	0.68	0.39–1.13	0.153	−0.44	0.28	0.64	0.36–1.08	0.112
AIC: 717.96. AUC ROC: 0.63.													

Note. Logistic regression based on Attitudes II. *p*-value < 0.05.

**Table 4:** Logistic regression to predict psychological support based on perceptions.

Variables			Univariate Analysis						Multivariate Analysis (Optimal Model)				
			*n*	β	EE	OR	CI 95%	*p*	β	EE	OR	CI 95%	*p*
Demographics	Intercept								−2.37	0.64	0.09	0.02–0.31	<0.001
	Gender: Male		783	−0.12	0.23	0.89	0.56–1.38	0.615	-	-	-	-	-
	Age		783	−0.02	0.01	0.98	0.96–0.99	0.006	−0.02	0.01	0.98	0.96–1	0.035
	Contracting COVID-19		783	0.72	0.29	2.06	1.14–3.59	0.013	0.85	0.32	2.34	1.22–4.35	0.008
Perceptions	Because of my job I feel emotionally exhausted	DAILY	1 (ref)						1 (ref)				
		WEEKLY	783	0.97	0.51	2.65	1.04–8.15	0.058	1.04	0.53	2.82	1.07–8.88	0.05
		MONTHLY	783	1.5	0.48	4.48	1.90–13.16	0.002	1.7	0.51	5.47	2.17–16.90	<0.001
		INFREQ.	783	2.51	0.48	12.34	5.26–36.23	<0.001	2.54	0.54	12.71	4.76–40.93	<0.001
	Through my work I feel that I am positively influencing other people’s lives	DAILY	1 (ref)						1 (ref)				
		WEEKLY	783	0.05	0.21	1.05	0.69–1.59	0.817	−0.16	0.24	0.85	0.53–1.35	0.499
		MONTHLY	783	0.98	0.3	2.65	1.44–4.77	0.001	0.83	0.34	2.29	1.16–4.45	0.015
		INFREQ.	783	0.49	0.43	1.63	0.67–3.61	0.249	0.34	0.48	1.4	0.53–3.45	0.477
	At work I feel that I am at the limit of my possibilities	*DAILY*	1 (ref)						1 (ref)				
		WEEKLY	783	−0.07	0.28	0.93	0.53–1.6	0.794	−0.72	0.31	0.49	0.26–0.88	0.018
		MONTHLY	783	0.67	0.24	1.95	1.23–3.12	0.005	−0.31	0.29	0.73	0.41–1.29	0.28
		INFREQ.	783	1.41	0.31	4.08	2.21–7.5	<0.001	0.1	0.39	1.11	0.51–2.37	0.795
	Despite everything, the pandemic has not worn out my vocation. If I could go back, I would choose my profession again	DAILY	1 (ref)						1 (ref)				
		WEEKLY	783	0.63	0.25	1.87	1.13–3.03	0.013	0.48	0.28	1.62	0.92–2.79	0.087
		MONTHLY	783	0.91	0.28	2.49	1.42–4.25	0.001	0.71	0.31	2.03	1.09–3.72	0.023
		INFREQ	783	0.3	0.31	1.35	0.71–2.44	0.336	−0.13	0.36	0.88	0.42–1.74	0.721
AIC: 667.74. AUC ROC: 0.74.													

Note. Logistic regression based on perceptions. *p*-value < 0.05.

**Table 5:** Working perceptions and attitudes towards work of the health professionals according to whether or not they required psychological help.

	Psychological Support Needed *n* = 139		Psychological Support not Needed *n* = 644		*t*	*p*
	Mean	SD	Mean	SD		
Attitudes II	5.41	1.73	5.93	1.70	3.265	0.001
Perceptions	42.96	7.24	47.98	8.18	6.690	<0.001

Note. Student’s *t*-test. *p*-value < 0.05.

**Table 6:** Binary logistic regression on the factor influencing the need for psychological support to the greatest extent.

Reference		B	Standard Error	Wald	Sig	OR	95% C.I. for EXP(B)	
							Inferior	Superior
Ref: No	Have you been transferred to other services to meet pandemic-related needs? (Yes)	0.508	0.200	6.482	0.011	1.663	1.124	2.459
	Constant	−0.339	0.324	1.092	0.296	0.713		

Note. Chi-squared test: 57.790. Degrees of Freedom: 10. Significance: 0.000. Cox–Snell’s R-squared: 0.071. Nagelkerke’s R-squared: 0.117.

## Data Availability

The data presented in this study are available on request from the corresponding author. The data is not publicly available because it belongs to a doctoral thesis not yet defended.

## Appendix A

**Table A1:** Personal–occupational description of the sample according to whether or not they required psychological help, and the variables related to the COVID-19 pandemic.

Sociodemographic Variables		Psychological Support Needed *n* = 139 (%)	Psychological Support not Needed *n* = 644 (%)	*p*
Gender	Male	28 (20.1)	148 (22.6)	0.319
	Female	111 (79.7)	496 (77.4)	
Age	<30 years	31 (22.3)	113 (17.5)	0.014
	31–50 years	88 (63.3)	365 (56.7)	
	>51 years	20 (14.4)	166 (25.8)	
Profession	Physician	27 (19.4)	110 (17.1)	0.766
	Nurse	71 (51.1)	332 (51.6)	
	Physiotherapist	4 (2.9)	29 (4.5)	
	Assistant nurse	34 (24.5)	150 (23.3)	
	Specialized technicians *	3 (2.2)	23 (3.6)	
Time working in healthcare	<5 years	37 (26.6)	151 (23.4)	0.247
	6–10 years	19 (13.7)	86 (13.4)	
	11–15 years	22 (15.8)	87 (13.5)	
	16–20 years	24 (17.3)	91 (14.1)	
	21–25 years	17 (12.2)	92 (14.3)	
	26–30 years	13 (9.4)	56 (8.7)	
	>30 years	7 (5.0)	81 (12.6)	
**Variables related to the COVID-19 pandemic**		**Psychological Support Needed** ***n* = 139 (%)**	**Psychological Support Not Needed** ***n* = 644 (%)**	***p***
Have you contracted COVID-19? (GE)	Yes	19 (13.7)	46 (7.1)	0.012
	No	120 (86.3)	598 (92.9)	
Have you gone through a process of home isolation at some point during the pandemic (either due to contact with positive people or due to compatible symptoms)? (GE)	Yes	76 (54.7)	286 (44.4)	0.018
	No	63 (45.3)	358 (55.6)	
Have your vacation time or days off been denied or postponed at any point during the pandemic? (GE)	Yes	73 (52.5)	280 (43.5)	0.032
	No	66 (47.5)	364 (56.5)	
Have you been transferred to other services to deal with the contingencies derived from the pandemic? (GE)	Yes	60 (43.2)	210 (32.6)	0.012
	No	79 (56.8)	434 (67.4)	

Note. Chi-squared test (χ^2^). *p*-value < 0.05. * Includes emergency medical technicians, radiology technicians and laboratory technicians. GE: Items recommended and approved by the group of experts.

**Table A2:** Beliefs and attitudes (I and II) of the health professionals according to whether or not they required psychological help (in %).

Beliefs	Psychological Support Needed (%)			Psychological Support Not Needed (%)			*p*
	Yes	No	I Do Not Know	Yes	No	I Do Not Know	
Have you lacked the appropriate PPE at any time during the pandemic? (GE)	73.4	25.2	1.4	64.1	33.7	2.2	0.113
Do you think that having more staff would have helped reduce the work overload during the pandemic? (GE)	95.7	2.9	1.4	94.9	3.1	2.0	0.892
Do you think there will be a second “post-traumatic stress pandemic” among healthcare personnel due to the events experienced during the COVID-19 pandemic? (GE)	85.6	3.6	10.8	72.2	12.1	15.7	0.002
Do you think that the COVID-19 pandemic has highlighted the shortcomings of the healthcare system? (GE)	95.7	4.3	0.0	93.9	5.0	1.1	0.439
**ATTITUDES I STATEMENTS** *During this pandemic, with what probability would you* *be more predisposed to work in the following situations?*	**Probable**	**Not** **Probable**	**I Do Not Know**	**Probable**	**Not Probable**	**I do Not Know**	***p***
If a family member died from COVID-19.	50.4	27.6	22.0	57.2	17.2	25.6	0.026
**ATTITUDES II STATEMENTS** *Do you agree or disagree with the following statements?*	**Agree**	**Disagree**		**Agree**	**Disagree**		***p***
All healthcare workers have a duty to work during the pandemic, even if there is an increased risk to their health.	43.2	56.8		53.7	46.3		0.015
Those who refuse to work during this health crisis should be sanctioned in some way.	36.0	64.0		46.7	53.3		0.013
My obligation to my job is above my family duties.	15.1	84.9		23.1	76.9		0.022
I would be willing to voluntarily give up my days off or vacation time to continue working if required by my unit.	41.0	59.0		55.4	44.6		0.001

Note. Chi-squared test (χ^2^). *p*-value < 0.05. GE: Items recommended and accepted by the group of experts.

**Table A3:** Working perceptions of the health professionals according to whether or not they required psychological help, and correlations with the ordinal and frequency variables using Kendall’s Tau B.

Perceptions	Psychological Support Needed *n* = 139 (%)				Psychological Support not Needed *n* = 644 (%)						*p*
	Infrequently	Monthly	Weekly	Daily	Infrequently	Monthly	Weekly	Daily	Tau-B	Gamma	
Because of my job I feel emotionally exhausted (MBI)	3.6	14.4	35.3	46.8	17.4	26.2	38.0	18.3	0.245	0.523	<0.001
I feel physically and psychologically exhausted when I leave work (MBI)	2.9	15.1	36.7	45.3	13.5	25.3	39.0	22.2	0.203	0.443	<0.001
I fell “burned out” by my current job (GE)	13.7	20.9	36.0	29.5	29.2	29.2	26.4	15.2	0.177	0.372	<0.001
Since the pandemic started, I have a hard time sleeping at night (FCV)	20.9	23.7	33.8	21.6	37.7	27.5	26.7	8.1	0.171	0.359	<0.001
Through my work I feel that I am positively influencing other people’s lives (MBI)	5.8	14.4	30.2	49.6	4.0	6.2	32.9	56.8	0.075	0.173	0.039
At work I feel that I am at the limit of my possibilities (MBI)	25.9	18.0	38.1	18.0	37.4	28.0	28.3	6.4	0.152	0.322	<0.001
I have been afraid of being infected while working (FCV)	15.1	15.1	30.9	38.8	19.6	21.1	30.3	29.0	0.082	0.172	0.076
My biggest fear is infecting my loved ones (FCV)	7.2	15.8	12.9	64.0	6.5	11.3	18.0	64.1	0.013	0.032	0.717
Despite everything, the pandemic has not worn out my vocation. If I could go back, I would choose my profession again (GE)	10.8	16.5	20.1	52.5	10.2	8.5	13.8	67.4	0.104	0.238	0.003
My institution adequately values my work as a healthcare professional (FR)	56.1	18.0	15.8	10.1	42.9	23.0	21.6	12.6	0.086	0.198	0.009
I feel supported by my direct superiors (FR)	35.3	21.6	25.9	17.3	23.9	25.8	26.1	24.2	0.080	0.171	0.015
There is potential for career advancement in my current position (FR)	69.8	17.3	8.6	4.3	67.9	14.8	10.4	7.0	0.024	0.065	0.459
I don´t have time to finish all my tasks during the working day (FR)	20.1	25.2	33.1	21.6	28.9	24.1	30.6	16.5	0.070	0.151	0.029
In my work I am very satisfied (FR)	12.9	24.5	43.2	19.4	5.4	18.5	40.1	36.0	0.146	0.317	<0.001
I manage to disconnect from work in my free time (FR)	17.3	30.9	38.1	13.7	16.0	21.1	40.1	22.8	0.083	0.180	0.009
I consider that my work is useful to society (GE)	2.2	2.9	19.4	75.5	1.1	3.4	16.0	79.5	0.036	0.107	0.325
Society recognizes my work as a health professional (GE)	36.7	34.5	21.6	7.2	34.3	29.2	22.0	14.4	0.052	0.113	0.099
I have felt discriminated against by my social environment (family or friends) due to a fear of infection (GE)	64.0	15.1	17.3	3.6	67.1	15.2	14.8	3.0	0.027	0.069	*0.439*

Note. Ordinal test. Kendall’s Tau-B and Gamma. *p*-value *<* 0.05. GE: Items recommended and accepted by the group of experts. MBI: Items derived from the Maslach Burnout Inventory. FCV: Items derived from the COVID-19 Fear Scale. FR: Items derived from the Font-Roja job satisfaction scale.
